# Inflammatory and Humoral Immune Response during Ebola Virus Infection in Survivor and Fatal Cases Occurred in Sierra Leone during the 2014–2016 Outbreak in West Africa

**DOI:** 10.3390/v11040373

**Published:** 2019-04-23

**Authors:** Francesca Colavita, Mirella Biava, Concetta Castilletti, Simone Lanini, Rossella Miccio, Gina Portella, Francesco Vairo, Giuseppe Ippolito, Maria Rosaria Capobianchi, Antonino Di Caro, Eleonora Lalle

**Affiliations:** 1 National Institute for Infectious Diseases “L. Spallanzani”, IRCCS, Via Portuense 292, 00149 Rome, Italy; francesca.colavita@inmi.it (F.C.); mirella.biava@inmi.it (M.B.); simone.lanini@inmi.it (S.L.); francesco.vairo@inmi.it (F.V.); giuseppe.ippolito@inmi.it (G.I.); maria.capobianchi@inmi.it (M.R.C.); antonino.dicaro@inmi.it (A.D.C.); eleonora.lalle@inmi.it (E.L.); 2 EMERGENCY-NGO, Via Santa Croce 19, 20122 Milan, Italy; rossella.miccio@emergency.it (R.M.); gina.portella@emergency.it (G.P.)

**Keywords:** ebola virus, sierra leone, immune response, inflammation, cytokines, antibody

## Abstract

Ebola virus (EBOV) infection is characterized by an excessive inflammatory response, a loss of lymphocytes and a general paralysis of the immune system, however pathophysiological mechanisms are not fully understood. In a cohort of 23 fatal and 21 survivors of ebola virus disease (EVD) cases admitted to the Emergency Ebola-Treatment-Center in Goderich (Freetown, Sierra Leone) during the 2014 to 2016 EBOV epidemic in Western Africa, we analyzed the pathway-focused gene expression profile of secreted proteins involved in the immune response and the levels of specific anti-EBOV IgM and IgG from the time of admission till discharge or death. We observed a dysregulated inflammatory response in fatal patients as compared to survivors, mainly consisting of the upregulation of inflammatory mediators, whose extent directly correlated with viremia levels. The upregulation persisted and intensified during the late phase of infection. Relevant differences were also found in humoral immunity, as an earlier and more robust EBOV antibody response was observed in survivor patients.

## 1. Introduction

Ebola virus (EBOV) is a member of the *Filoviridae* family and is classified in the genus Ebolavirus, species Zaire ebolavirus. EBOV is responsible for a devastating viral hemorrhagic fever known as Ebola Virus Disease (EVD) and so far, is the most lethal species among the ebola viruses known to be pathogenic for humans (case-fatality rate up to 90%) [1]. Recently, a new ebolavirus species was isolated in bats in Sierra Leone, the Bombali ebolavirus, even if, as Reston ebolavirus, it has not yet been shown to cause disease in humans [2]. EBOV caused numerous human epidemics since its first isolation in 1976, including the new declared outbreak ongoing in the North Kivu Province of the Democratic Republic of the Congo [3]. The epidemic in West Africa in 2014 to 2016 represents one of the most dramatic infectious emergencies of the past decades, with unique magnitude (28,646 cases and 11,323 deaths reported) and multi-country spread [4].

Despite its impact on human health, EVD pathogenesis is still incompletely understood. Much of what is known has been acquired through studies on in vitro infections and on non-human primates (NHPs).

The failure of the immune response in controlling viral replication involves both the innate and adaptive immune system [5,6,7]. The innate immune reaction to EBOV is characterized by a “cytokine storm”, with the secretion of numerous pro-inflammatory cytokines, including IL-1β, IL-6, IL-8, CCL2, CCL3, CCL4, which induce a huge number of immune mediators and may contribute to the impairment of the vascular system, disseminated intravascular coagulation, and massive loss of innate and adaptive immune cells [8,9]. This scenario was observed in the plasma of humans following EBOV infection, even if the majority of information about the human immune response concerns past epidemics with limited sample sizes and rare longitudinal sample collection [10,11]. Indeed, studies are constrained by the requirements of maximum bio-containment measures and difficulty in obtaining samples at multiple time points throughout the course of the disease in an outbreak scenario. To our knowledge, only one recent study described the kinetics of the expression of soluble inflammatory mediators, in a longitudinal blood samples collection from 180 hospitalized patients with EVD treated in Guinea, concluding that the control of endothelial and gastric integrity, as well as T-cell immunity, correlated with EVD survival [12]. Profound suppression of adaptive immune response has also been observed, including impaired humoral response and T lymphocyte functional exhaustion and apoptosis [7,13,14]. Previous studies report that the natural serologic response consisted of EBOV-specific IgM detected as early as two days since symptom onset (DSO), but occurring 10–29 DSO in most patients; and specific IgG detected as early as 6 DSO, but occurring 6–18 DSO in most individuals, suggesting classic kinetics of an IgM response before the IgG response [11,15]. In addition, the humoral response to EBOV infection was reported as absent or diminished in fatal cases, while survivors demonstrated the presence of significant levels of virus-specific IgM and IgG followed by the activation of cytotoxic cells at the time of antigen clearance from the blood [16]. Notwithstanding, the antibody response in EVD patients is still controversial and studies on this aspect are rare [17,18,19,20]; therefore, defining a comprehensive profile of the immune response is essential for the effective management of patients and countermeasure development.

We investigated the gene expression profile of lymphokines/interleukins and chemokines and the levels of specific anti-EBOV IgM and IgG in fatal and survivor patients admitted during the 2014 to 2016 EBOV outbreak at the Emergency Ebola Treatment Center (ETC) in Goderich (Freetown, Sierra Leone) and sampled at the time of admission and longitudinally until discharge or death. The study lacked a healthy control group due to the constraints of the field settings. The comparison was made within the EVD-positive patients thus narrowing the inferences of our observations.

## 2. Materials and Methods

### 2.1. Study Group

Leftover diagnostic plasma samples from 44 patients who tested EVD-positive at the Italian Laboratory at the Emergency ETC in Goderich (Freetown, Sierra Leone) during the 2014 to 2016 outbreak, were included in the study [21]. Of the 44 EVD-positive patients, 25 (56.8%) were female and the median age was 30 years (interquartile range, 39.7–17 years); 23 had a fatal outcome (fatal group, 14 female) and 21 a positive outcome (survivor group, 11 female) (Table S1). All samples were collected during the hospitalization in a range of 3–15 (median: 7.5) DSO and stored (anonymized and identified as fatal or survivor) at −20 °C until the beginning of research activities carried out at the Princess Christian Maternity Hospital (PCMH) in Freetown and at the Emergency Surgical and Pediatric Hospital in Goderich. All 44 patients were included in the cytokine gene expression study and for 32 of them, two consecutive samples were analyzed (mean ± Standard Deviation [SD] interval between the two longitudinal sampling was 5 ± 2.5 days), accounting for a total of 75 samples tested. Samples collected <7 DSO were considered “acute phase samples” (*n* = 42 with a mean Log cp EBOV-RNA/mL: 8.0 ± 0.8: fatal = 23, mean Log cp EBOV-RNA/mL: 8.3 ± 0.8; survivor = 19, mean Log cp EBOV-RNA/mL: 7.6 ± 0.7); samples collected >8 DSO were considered “late phase samples” (*n* = 33 with a mean Log cp EBOV-RNA/mL: 5.8 ± 2.1, fatal = 15 with a mean Log cp EBOV-RNA/mL: 7.6 ± 1.5; survivor = 18, mean Log cp EBOV-RNA/mL: 4.4 ± 1.0) (Table S1). The cut off for early versus late was set to 7 DSO based on previous evidence that this showed that the viral load turning point between patients who survived or succumbed, with a decline for patients who survived and a steady increase for patients who succumbed [19,22,23].

In addition, 13 fatal and 13 survivor EVD-patients were analyzed to address the humoral immune response (anti-EBOV IgM and IgG), using 2 to 7 sequential plasma samples, collected 1 to 3 days apart in a timeframe ranging from 2 and 16 DSO, accounting for a total of 137 plasma samples. The study protocol was approved by the Ethical Committee of Sierra Leone.

### 2.2. Cytokines Gene Expression

**Figure 1:**
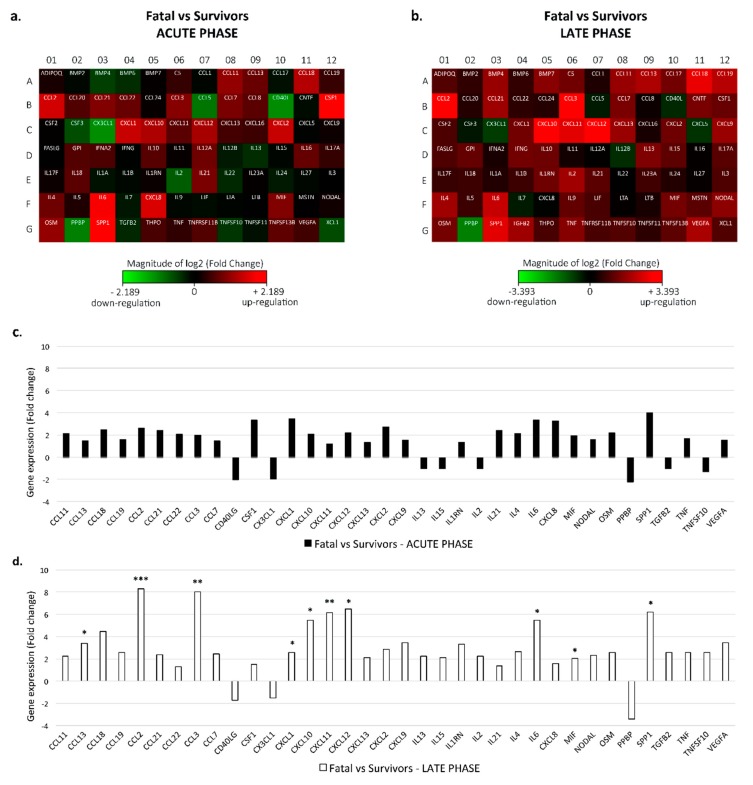
mRNA levels of 84 cytokine in EBOV-fatal compared to EBOV-survivor patients during the acute and late phases of infection. (**a**,**b**) Heat-maps of the fold change in mRNA levels of a panel of cytokines in 23 EBOV-fatal samples as compared to 19 EBOV-survivor samples during the acute phase (**a**); and 15 EBOV-fatal samples as compared to 18 EBOV-survivor samples during the late phase (**b**) of infection. Fold changes ≥2 (upregulation, red) or <−2 (downregulation, green) are shown. (**c**,**d**) Up- or down-regulation of cytokines mRNA levels in fatal versus survivor EVD patients during the acute (**c**) and late (**d**) phases of infection. The data are reported as positive (up-regulation) or negative (down-regulation) mean fold changes observed during the acute (black bars) and late phase of infection (white bars). Only cytokines for which the fold change was more than 2 (up-regulation) or less than −2 (down-regulation) were considered. Significant statistical differences in mRNA levels (∆Ct) between the two groups are reported (* *p* < 0.05, ** *p* < 0.01, *** *p* < 0.001 in Mann–Whitney test).

Total RNA extraction was performed using QIAamp^®^ Viral RNA Mini Kit (Qiagen, Hilden, Germany) following manufacturer’s instructions and part of the extracted RNA was tested for EBOV RNA and the residual was stored at −20 °C. Viral genome amplification was performed using a qPCR assay (RealStar Filovirus Screen RT-PCR 1.0 kit, Altona Diagnostics, Hamburg, Germania) as described elsewhere [21]. cDNA synthesis was subsequently performed from 0.5 µg of RNA using the RT2 First Strand Kit (Qiagen), following manufacturer’s instructions. The DNAse treatment step for genomic DNA elimination was included in the protocol. The RNA quality of all the samples was checked through amplification of a house-keeping gene (RNaseP) in qRT-PCR. Gene expression profiling was done with qRT-PCR, using the RT^2^ Profiler™ PCR Array Human Cytokines & Chemokines (SuperArray PAHS-150 Kit; Bioscience Corporation, Frederick, MD, USA) in 96-wells plate format. The system was pathway-focused, profiling the expression of 84 key secreted proteins involved in the immune response (detailed in the legend of Figure 1 and Table S2). Nucleic acid amplification was performed on ABI Prism 7900HT (Applied Biosystems, Foster City, CA, USA).

For each sample, ABI Prism 7900 Sequence Detection System (SDS) software plotted an amplification curve by relating the fluorescence signal intensity (∆Rn) to the cycle number. The PCR results were analyzed using the PCR Array Data Analysis Web Portal (Bioscience Corporation). We performed a relative quantitative analysis, using the 2^(−∆∆*C_t_*) value, where ∆*C_t_* = *C_t_* (target) − *C_t_* (endogenous control) and ∆∆*C_t_* = ∆*C_t_* (sample) − ∆*C_t_* (calibrator). *β-actin* was used as a housekeeping gene. At the end, in the absence of a healthy control group, the fold change (2^(−ΔΔ*C_t_*)) of the fatal group versus the survivor group was calculated with default software option. Upregulation or downregulation of gene expression was considered reliable and relevant when the fold change was ≥2 or <−2, respectively, as recommended in the PCR Array Data Analysis Web Portal. Descriptive statistics were performed as described below.

### 2.3. Humoral Response Analysis

Indirect immunofluorescence assay (IFA) was performed to evaluate the anti-EBOV specific antibodies. The in-house slides were prepared at the INMI BSL-4 laboratory in Rome, using Vero E6 infected with EBOV isolate (Ebola Zaire H. sapiens tc/gin/2014/Gueckedou-C05, kindly provided by S. Gunther); 24 hours (h) post infection cells were trypsinized and mixed with uninfected Vero E6 in 1:1 proportion, washed twice in Dulbecco’s Phosphate-Buffered Saline 1× (Sigma-Aldrich, St. Louis, MO, USA) and fixed for 30 min in acetone at −20 °C. We preliminarily tested the EBOV home-made slides with three negative and three positive control samples at different 2-fold dilutions (from 1:2 to 1:40): the 1:20 dilution resulted in the best condition to eliminate the background signal and obtain a clear positive fluorescence with a good sensitivity. The slides were then shipped to Freetown (Sierra Leone) under controlled temperature.

To detect anti-EBOV-IgM, plasma samples were pre-treated with Eurosorb (Euroimmun, Lubecca, Germany) for 30 min at room temperature (RT), centrifuged at 3500 rpm for 10 min and diluted (screening dilution: 1:20) in normal saline (NS, Fresenius Kabi, Bad Homburg vor der Höhe, Germany) solution. For anti-EBOV-IgG tests, plasma samples were directly diluted 1:20 in NS. Each plasma sample was serially diluted from 1:20 down to 1:1280 to estimate the antibodies titre and incubated for 1 h at RT. The slides were washed with NS and incubated for 1 h RT with anti-human IgM and IgG rabbit antibodies conjugated with FITC and counterstained with Evans Blue (Euroimmun, Lubecca Germany). The slides were finally inactivated in 2% paraformaldehyde solution. For biosafety reasons, all the aforementioned procedures were performed in a class III biosafety cabinet. PBS-glycerol 1% was used as mounting media (Euroimmun, Lubecca, Germany). The results were analyzed with the fluorescence microscope Nikon Eclipse E200. In each experiment, anti-EBOV IgM- and -IgG positive controls and a negative control were included. As a quality check, the slides were tested both in Italy and in Sierra Leone before they were shipped with anti-EBOV IgM- and IgG -positive control samples and stored at −20 °C.

### 2.4. Statistical Analysis

The Mann–Whitney test was used to evaluate the differences in the cytokines mRNA levels between the fatal and survivors groups in the acute and late phase of infection. The Spearman correlation test was used to evaluate the correlation between cytokine mRNA levels and between EBOV viremia and anti-EBOV IgM/IgG. The LogRank test was used for the evaluation of the Kaplan-Meier curves of anti-EBOV specific IgM/IgG in survivor and fatal groups. The statistical analysis was performed using GraphPad Prism and STATA 13.1 statistical packages.

## 3. Results

### 3.1. Cytokine Expression

To study the inflammatory response in fatal and surviving EVD patients, we examined the expression of a focused panel of key cytokines and chemokines both during acute and late phases of infection. We only analyzed EVD-positive samples, as no healthy control group was available for this field study, and no replicates were performed due to constraints in the outbreak and resources-limited setting in which samples were collected.

The heat-maps in Figure 1a,b show the fold-changes of the cytokines mRNA levels in acute and late samples from EVD fatal patients versus EVD survivors, respectively (see also Table S2).

During the acute phase, there were no significant (2-fold) differences in the mRNA expression levels of immune mediators in fatal patients versus survivors. Moreover, of the 84 analyzed factors, 16 (19%) showed ≥2-fold variation in gene expression in fatal as compared to the survivor group, specifically an upregulation of up to >3/4-fold was seen for *CSF1*, *CXCL1*, *IL-6*, *CXCL8* and *SPP-1* cytokines (Figure 1a–c).

On the contrary, in the late phase of disease, an increased dysregulation in inflammatory response was observed in the fatal group as compared to survivors. In particular, a higher number of genes (36%) showed a ≥2-fold increase in gene expression in fatal versus survivor patients (Figure 1b). Among these cytokines, we found a statistically significant up-regulation in late fatal as compared to late survivors for the following mediators: The chemokines *CCL13*, *CCL2*, *CCL3*, *CXCL1*, *CXCL10*, *CXCL11*, *CXCL12*; the pro-inflammatory factor IL6; the pro-inflammatory lymphokine MIF and the growth factor *SPP1* (Figure 1d). In addition, also the extent of gene expression variation was increased at this stage of infection (i.e., up to >8-fold change seen for the inflammatory chemokines *CCL2* and *CCL3*) as compared to the acute phase, where a maximum of 3-fold variation was observed (Figure 1c). The only mediator showing a significant down-regulation in fatal versus survivor patients during both phases (more intense in the latter) was the chemokine *PPBP*.

The correlation between the level of each cytokine mRNA with the corresponding EBOV viremia levels was evaluated. A statistically significant (*p* < 0.05) correlation was observed only in the fatal group during the late phase of infection (Table 1). In particular, a strong (*r* ≥ ± 0.6) positive correlation with EBOV viremia was observed in those genes which were mainly involved in leukocytes recruitment and activation. Among these, the chemokines *CCL2* and *CXCL12* resulted in high correlation values (*r* = 0.859 and = 0.8206 respectively; *p* < 0.0001), suggesting a link between the prolonged viral replication and the sustained signaling of chemotaxis for lymphocytes and macrophages and prolonged status of inflammation. Finally, *PBPP* was again the only gene shown to be negatively correlated to viral RNA levels (*r* = −0.621; *p* = 0.0103).

### 3.2. Anti-EBOV IgM and IgG Response

The humoral response against EBOV infection was evaluated on a longitudinal collection of plasma samples (total number: 94) collected from 26 EVD-infected patients (13 fatal and 13 survivors) and analyzed in relation to DSO in a timeframe between 2 and 16 DSO.

When analyzing the Ig profiles of the 26 patients, we observed that 18 (69.2%) of them developed both anti-EBOV IgM and IgG (survivors = 12, fatal = 6) and 5 (19.2%) presented IgG (4 patients, survivors = 1, fatal = 3) or IgM (1 fatal patient) only; 3 (11.5%) patients, all fatal, did not develop specific IgM nor IgG within the timeframe considered in the study.

**Figure 2:**
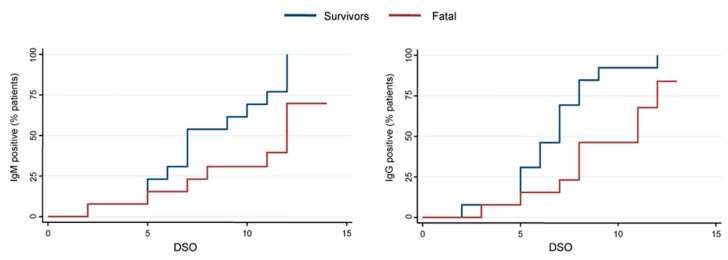
Kaplan–Meier curves reporting the proportion of survivor (blue line) and fatal (red line) patients developing anti-EBOV specific IgM (**left**) and IgG (**right**) in relation days from symptom onset (DSO). The proportion of patients is expressed as a percentage. Statistical significance (*p* < 0.05) by Log-rank test: IgM, *p* = 0.0351; IgG, *p* = 0.0141.

Kaplan–Meier curves are shown in Figure 2: The antibodies appearance was earlier and more intense in survivors than in fatal patients (*p* = 0.0141) with 100% of survivors developing anti-EBOV IgG within DSO 12. A similar trend was observed for the IgM response (*p* = 0.0351), although one survivor patient resulted IgM-negative (<1:20) for the whole timeframe considered in the study.

**Figure 3:**
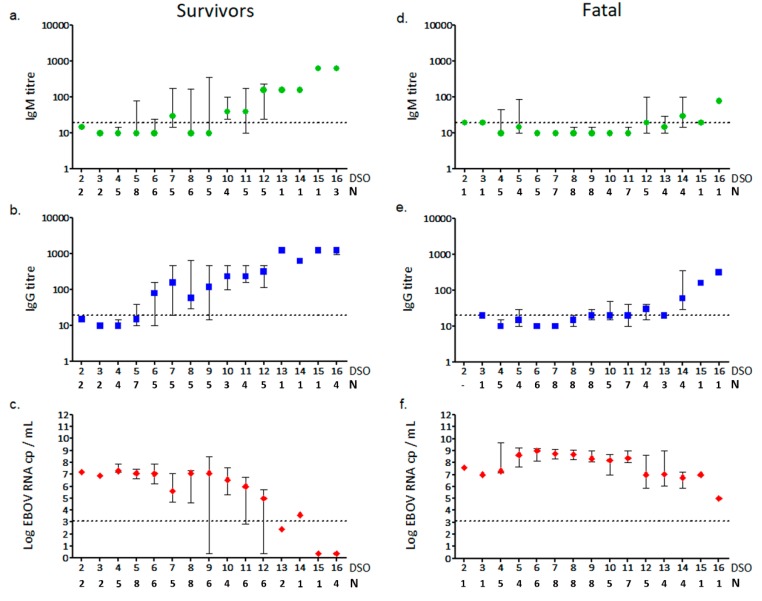
Trend of EBOV-specific antibody response and viremia in relation to DSO. IgM (**a**), IgG (**b**) titres and viral load (**c**) detected in survivor patients. IgM (**d**), IgG (**e**) titres and viral load (**f**) detected in fatal cases. Data are shown as median and interquartile range values of antibody titres (expressed as reciprocal of plasma dilution) and viral load (expressed as Log cp RNA/mL). Dotted-lines represent the limits of detection of IFA (1:20, in **a**, **b**, **d** and **e**) and viral load (3.11 Log cp RNA/mL, in **c** and **f**). DSO = days since symptoms onset; N = number of samples tested for each time-point.

The overall analysis of anti-EBOV IgM and IgG levels in relation to the onset of symptoms revealed that most of the patients included in this study developed a humoral response from DSO 7, with increasing antibodies titre at the end of the second week of illness, which corresponded to the progressive reduction of EBOV viremia, especially when survivors were considered (Figure 3, Table S3). Indeed, a significant inverse correlation between viral load and antibodies titre was obtained, with a stronger correlation value for IgG in survivors (*r* = −0.8207, *p* < 0.0001) (Figure 3b,c). Our results showed that anti-EBOV IgG response preceded the appearance of IgM, which in many cases was even absent. This result was more pronounced when the two groups were separately analyzed. In survivors, IgG was detected earlier than IgM, and started since DSO 5 in most of the cases; IgM levels remained moderate until DSO 11 (Figure 3a,b, Table S3). On the contrary, fatal cases showed a delayed anti-EBOV IgG response, not rising until DSO 8 in most of the cases (Figure 3d,e, Table S3).

Moreover, the levels of both antibody classes were remarkably different between the two groups: Anti-EBOV IgM titre remained low in fatal cases until the end of the second week after the onset of the diseases, with maximum values of 1:40–1:160, with respect to 1:320–1:640 detected in the survivors group in the same timeframe. In addition, almost 50% of fatal EVD patients did not develop IgM (<1:20) within DSO 16. Similar results were obtained for IgG: Anti-EBOV IgG titre did not exceed 1:320 within 16 DSO in the fatal group with a steady increase in viremia (Figure 3f), while higher titres (up to 1:1280) were observed in survivors as early as at 13 DSO (Table S3).

## 4. Discussion

In this paper, we described the gene expression profile of a panel of key chemokines and cytokines and the levels of specific anti-EBOV IgM and IgG in a cohort of fatal and survivor EVD patients infected during the recent EBOV outbreak that occurred in Sierra Leone in 2014 to 2016. We tested a precious collection of plasma samples from a large number of patients, obtained at the time of admission at the Emergency ETC in Goderich (Freetown, Sierra Leone) and longitudinally until their discharge or death.

Previous reports indicated that the early and vigorous cytokine storm was linked to an unfavorable prognosis of EVD patients [12,13,14,19]. Our study seems to support this idea and adds new insights. In fact, results from the gene expression profiling showed a higher dysregulation, consisting mainly in up-regulation, of the inflammatory mediators in EVD fatal cases as compared to the survivor group during the acute phase of the disease, although no significant differences were found for any of the chemokines and cytokine genes analyzed. Notably, the analysis in the late phase of infection highlighted that this dysregulation seemed to persist and intensify in fatal patients versus survivors, with statistically significant differences in a number of genes between the two groups. In addition, we observed that the levels of gene expression of inflammatory chemokines directly correlated with the viremia load, thus suggesting an association between high levels of virus replication, excessive and persistent status of inflammation and fatal outcome of EVD [12,14].

In agreement with previous studies, the dysregulation observed in the fatal group during the late phase of the infection mainly consisted in the upregulation of secreted inflammatory mediators, such as *CCL2*, *CCL3*, *CCL13*, *CXCL1*, *CXCL10*, *CXCL11*, *CXCL12*, *IL6*, *MIF*, *SPP1*, which are produced by myeloid cells and normally play a role in the defense mechanisms with the regulation of the adaptive immune response, specifically the migration of immune system cells associated with the trafficking of leukocytes in immune surveillance and inflammatory cell recruitment [24,25]. Furthermore, the dysregulation of these cytokines is normally involved in other inflammatory diseases, thus suggesting a direct role of the mediators and the immune cells in the pathogenesis. These mechanisms may also be reflected in EVD, representing an important source of inflammatory mediators [24]. Interestingly, we observed a persistent trend in the down-regulation of PPBP, which is a potent neutrophils chemoattractant and activator involved in platelet activation, but also a protein involved in the host defense against bacterial and fungal infections [26]; it is possible that there is a negative role in the progression of EBOV infection, to our knowledge never reported so far, which is corroborated also by the fact that we found its expression inversely correlated with viremia levels in fatal cases during the late phase of infection.

In comparison to other studies, no significant differences in the expression of other key inflammatory cytokines *TNF* or *IL1β* and interferon (*IFN-α* and −*γ*) genes were found between fatal versus survivors both in the acute and late phases of illness [11,16,17,27]. This discrepancy may reflect differences in sampling time, although other factors may be involved. Nevertheless, our findings suggest the idea that failure of the immune system to restore “normal” expression levels after the cytokine storm caused by virus replication/dissemination is the hallmark of progression towards fatal outcome of EVD.

The prolonged and significant excessive alteration of inflammatory cytokines levels in the fatal group may contribute to the EVD-typical severe multiple organ failure as a result of immune-mediated cell damage and may be linked to an ineffective adaptive immune response. In fact, the analysis of the humoral response showed that patients who survived the EBOV infection presented an early and robust antibody response against the virus with respect to patients who died. In line with previously reported evidence [16,19,28,29], we found that patients with fatal outcome presented lower, or often absent, levels of both EBOV-specific IgM and IgG, which, when detected, appeared later than in survivors. Our findings suggest that the classic kinetics of antibody response does not always occur during EBOV infection, independently from the clinical outcome. In fact, in our cohort of EVD cases IgG was detected before IgM, and in a large number of patients, the IgM response was not evidenced before 16 DSO. These data are consistent with previous published [19,29] and personal unpublished experiences.

The humoral host response kinetics in the context of EBOV infection should be further investigated in a larger cohort of patients to clarify diagnostic interrogatives, set up clear diagnostic protocols, and correctly interpret epidemiological studies.

In the present study, IgG titres reached high values as early as 6 DSO in individuals who survived the infection, and this clearly concurred with the dropping of viremia. This highlights that IgG levels during the first week of symptoms may predict an effective control of the infection by the host immune system and thus could represent a marker for a positive progression and outcome of the disease [30]. The longitudinal analysis represents an important value to the description of the immune response established during natural EBOV infection and, to our knowledge, the present report is one of the largest studies investigating the antibody response during the course of EBOV infection [15,17,29].

This study has some limitations. Firstly, the type of biological samples chosen for mRNAs extraction in the gene expression analysis, as plasma samples are not the ideal ones. However, a recent study evaluated the transcriptome profile in samples collected in Guinea [31] and experimental evidences demonstrating the presence of extracellular RNAs in plasma [32,33]. Furthermore, all specimens used in the present study were screened for quality, tested for the human *RNaseP* gene, providing similar Ct values in all samples (average Ct value: 29.5 ± 2.5). In addition, the internal control present in the RT2 Profiler PCR Array Kit was always positive and the kit house-keeping gene (*β-actin*) had an average Ct value of 29.3 ± 2.47 in all samples. Secondly, the study lacked a healthy control group and no replicate testing could be performed on collected samples. This bias could not be avoided, samples were collected within an emergency context, and testing was performed in a field resources-limited setting, thus narrowing the experimental options that could be undertaken. However, there are very few papers performed in the field reaching the sample numerosity achieved in the present study; we believe that the data retrieved from this field experience can contribute to providing important insight into EVD progression and pathogenesis of the disease.

## 5. Conclusions

Our study seems to confirm previously published data showing that there are significant differences in the immune response between EVD fatal and survivor patients, both in antibodies and inflammatory mediators, which likely contribute to the outcome of the disease. Our study suggests an important dysregulation of several cytokines in the fatal group as compared to the survivors in the late phase of infection showing high levels of pro-inflammatory mediators, which play a role in the immune system. This sustained and excessive alteration in the fatal group may be related to the failure to produce anti-EBOV immunoglobulins and finally, to the inability to control virus replication and disease progression in fatal outcomes.

## Figures and Tables

**Table 1:** Correlation between the fold changes in mRNA levels for the indicated cytokines and the corresponding EBOV viremia in patients from the fatal group during the late phase of the infection. Only cytokines with correlation coefficient (*r*) > 0.600 and *p* < 0.05 are shown. The only negative correlation is evidenced in bold character.

Gene	Correlation Coefficient (r)	95% Confidence Interval	*p*-Value
*BMP7*	0.694	0.288 to 0.889	0.003
*CCL11*	0.753	0.397 to 0.912	0.0008
*CCL13*	0.718	0.330 to 0.898	0.0017
*CCL18*	0.677	0.257 to 0.881	0.004
*CCL2*	0.859	0.622 to 0.952	<0.0001
*CCL21*	0.771	0.432 to 0.919	0.0005
*CCL3*	0.612	0.005 to 0.809	0.043
*CSF1*	0.700	0.298 to 0.891	0.0025
*CXCL12*	0.821	0.536 to 0.938	<0.0001
*CXCL13*	0.644	0.202 to 0.868	0.0071
*GPI*	0.700	0.298 to 0.891	0.0025
*IL10*	0.612	0.151 to 0.854	0.012
*IL12A*	0.641	0.191 to 0.867	0.007
*IL15*	0.688	0.277 to 0.886	0.0032
*IL18*	0.685	0.272 to 0.885	0.0034
*IL23A*	0.527	0.025 to 0.816	0.036
*IL3*	0.635	0.188 to 0.864	0.008
*MIF*	0.724	0.341 to 0.900	0.0015
***PPBP***	**−0.621**	**−0.858 to −0.165**	**0.0103**
*SPP1*	0.668	0.242 to 0.878	0.005
*TNF*	0.709	0.314 to 0.895	0.0021
*TNFRSF11B*	0.624	0.169 to 0.859	0.0099

## Supplementary Materials

The following are available online at https://www.mdpi.com/1999-4915/11/4/373/s1, Table S1: Characteristics of EVD patients, Table S2. Fold-changes in the pathway-focused gene expression of the single secreted proteins, Table S3. Kinetics of the anti-EBOV antibodies response and viremia in EVD fatal and survived patients.
