# Supplementary material for: Inflammatory and Humoral Immune Response during Ebola Virus Infection in Survivor and Fatal Cases Occurred in Sierra Leone during the 2014–2016 Outbreak in West Africa
Source: Viruses. 2019 Apr 23;11(4):373. doi: 10.3390/v11040373 (PMC6520887; doi:10.3390/v11040373)
Supplement: Supplementary file 1 [file viruses-11-00373-s001.zip › Table_S1.pdf]

Table S1. Characteristics of EVD patients.

Total (n=44)				
**Age** (Mean ±SD; Median (range))	30 ±16.7; 28.5 (2-72)			
**Sex** (% female)	56.8%			
**Range of DSO** (Median)	3-15 (7,5)			
**Log EBOV RNA cp/mL**	**Acute**		**Late**	
	8.0±0.8		5.8±2.1	
	**Survivors (n=21)**		**Fatal (n=23)**	
**Age** (Mean ±SD; Median (range))	31 ±17; 29 (7-72)		31.1 ±16.8 ; 29 (2-65)	
**Sex** (% female)	52.4%		60.9%	
**Range of DSO** (Median)	1-14 (4)		1-8 (4)	
**Log EBOV RNA cp/mL**	**Acute**	**Late**	**Acute**	**Late**
	7.6±0.7	4.4±1.0	8.3±0.8	7.6±1.5
