# Supplementary material for: Inflammatory and Humoral Immune Response during Ebola Virus Infection in Survivor and Fatal Cases Occurred in Sierra Leone during the 2014–2016 Outbreak in West Africa
Source: Viruses. 2019 Apr 23;11(4):373. doi: 10.3390/v11040373 (PMC6520887; doi:10.3390/v11040373)
Supplement: Supplementary file 1 [file viruses-11-00373-s001.zip › Table_S2.pdf]

Table S2. Fold-changes in the pathway-focused gene expression of the single secreted proteins.

A – Panel of analyzed genes

	01	02	03	04	05	06	07	08	09	10	11	12
**A**	ADIPOQ	BMP2	BMP4	BMP6	BMP7	C5	CCL1	CCL11	CCL13	CCL17	CCL18	CCL19
**B**	CCL2	CCL20	CCL21	CCL22	CCL24	CCL3	CCL5	CCL7	CCL8	CD40LG	CNTF	CSF1
**C**	CSF2	CSF3	CX3CL1	CXCL1	CXCL10	CXCL11	CXCL12	CXCL13	CXCL16	CXCL2	CXCL5	CXCL9
**D**	FASLG	GPI	IFNA2	IFNG	IL10	IL11	IL12A	IL12B	IL13	IL15	IL16	IL17A
**E**	IL17F	IL18	IL1A	IL1B	IL1RN	IL2	IL21	IL22	IL23A	IL24	IL27	IL3
**F**	IL4	IL5	IL6	IL7	CXCL8	IL9	LIF	LTA	LTB	MIF	MSTN	NODAL
**G**	OSM	PPBP	SPP1	TGFB2	THPO	TNF	TNFRSF11B	TNFSF10	TNFSF11	TNFSF13B	VEGFA	XCL1

B – Fold-changes of the mRNA levels in acute samples from EVD fatal patients versus EVD survivors

	1	2	3	4	5	6	7	8	9	10	11	12
**A**	1,1	1,2	-1,3	-1,1	1,3	1,3	1,6	2,1	1,4	1,3	2,5	1,6
**B**	2,6	1,3	2,4	2,0	1,4	2,0	-1,9	1,5	1,6	-2,0	1,2	3,3
**C**	1,1	-1,2	-2,0	3,5	2,1	1,2	2,2	1,3	1,6	2,8	-1,3	1,5
**D**	1,3	-1,1	1,8	1,1	1,6	-1,2	1,9	-1,6	-1,0	-1,0	1,1	1,5
**E**	1,2	1,8	1,2	1,4	1,3	-1,1	2,4	1,1	1,2	1,2	1,1	1,2
**F**	2,1	1,5	3,3	-1,1	3,2	1,4	1,3	1,0	1,0	1,9	1,3	1,6
**G**	2,2	-2,2	4,0	-1,1	1,4	1,7	1,9	-1,3	1,2	2,0	1,5	-1,1

C – Fold-changes of the mRNA levels in late samples from EVD fatal patients versus EVD survivors

	01	02	03	04	05	06	07	08	09	10	11	12
**A**	1,2	-1,3	2,0	1,2	1,9	1,5	-1,3	2,3	3,4	1,3	4,5	2,6
**B**	8,3	1,7	2,4	1,3	1,4	8,0	-1,4	2,5	1,0	-1,7	1,9	1,5
**C**	1,4	-1,4	-1,6	2,6	5,5	6,2	6,4	2,1	1,2	2,8	-1,5	3,5
**D**	1,6	1,9	-1,2	1,4	1,4	1,3	-1,4	-1,4	2,3	2,1	1,3	1,6
**E**	1,7	1,2	1,2	1,9	3,3	2,2	1,4	-1,4	1,9	1,2	-1,0	1,9
**F**	2,6	1,9	5,5	1,2	1,6	1,3	1,1	1,2	1,2	2,1	1,3	2,3
**G**	2,6	-3,4	6,2	2,5	1,2	2,5	1,5	2,6	1,1	2,0	3,4	-1,0
