# Supplementary material for: Inflammatory and Humoral Immune Response during Ebola Virus Infection in Survivor and Fatal Cases Occurred in Sierra Leone during the 2014–2016 Outbreak in West Africa
Source: Viruses. 2019 Apr 23;11(4):373. doi: 10.3390/v11040373 (PMC6520887; doi:10.3390/v11040373)
Supplement: Supplementary file 1 [file viruses-11-00373-s001.zip › Table_S3.pdf]

Table S3. Kinetics of the anti-EBOV antibodies response and viremia in EVD fatal and survived patients.

**SURVIVORS**	**DSO**	**2**	**3**	**4**	**5**	**6**	**7**	**8**	**9**	**10**	**11**	**12**	**13**	**14**	**15**	**16**
**1**	IgM				1:80		1:320	1:320								
	IgG				1:40		1:320	1:1280								
	Viremia				6.5		4.2	<3.1								
**2**	IgM					<1:20			<1:20			1:160				1:640
	IgG					<1:20			<1:20			1:320				1:1280
	Viremia					8.2			8.5			5.3				<3.1
**3**	IgM			<1:20		1:20								1:160	1:640	
	IgG					1:160								1:640	1:1280	
	Viremia			7.3		7.4								3.6	<3.1	
**4**	IgM							<1:20	<1:20		1:40	1:160				1:640
	IgG								1:80		1:160	1:320				1:1280
	Viremia							7.1	6.8		6.2	5.3				<3.1
**5**	IgM		<1:20	<1:20	<1:20		1:20			1:160			1:160			
	IgG		<1:20	<1:20	<1:20		1:20			1:640			1:1280			
	Viremia		7.6	8.2	7.3		5.6			4.4			<3.1			
**6**	IgM	1:20		1:20	1:40	1:40		1:640								
	IgG	1:20		1:20	1:40	1:160		1:640								
	Viremia	8.2		7.6	7.3	5.3		<3.1								
**7**	IgM	<1:20	<1:20	<1:20	<1:20	<1:20	1:20	1:20		1:40	1:320	1:320				
	IgG	<1:20	<1:20	<1:20	<1:20	<1:20	1:20	1:40		1:320	1:320	1:640				
	Viremia	6.2	6.5	7.3	7.1	7.6	7.3	7.4		6.2	5.3	<3.1				
**8**	IgM			<1:20	<1:20	<1:20	<1:20				<1:20					
	IgG			<1:20	<1:20		1:160				1:640					
	Viremia			7.1	7.6	6.8	6.5				<3.1					
**9**	IgM				<1:20	<1:20			1:80							
	IgG					1:80			1:320							
	Viremia				7.1	6.2		4.7	3.9			<3.1				
**10**	IgM				<1:20		1:40									
	IgG				1:20		1:640									
	Viremia				6.8		5.6				<3.11					
**11**	IgM							<1:20	<1:20	<1:20	<1:20	<1:20				1:20
	IgG							1:80	1:160	1:160	1:160	1:160				1:640
	Viremia							7.1	7.3	6.9	6,0	4.7	4.4			<3.1
**12**	IgM							<1:20	<1:20	1:40	1:40	1:40				
	IgG							1:20	1:20			1:80				1:1280
	Viremia							8.5	8.0	8.2	7.3	6.2				<3.1
**13**	IgM				1:80											
	IgG				1:1280											
	Viremia				6.4			4.6	<3.1							

FATAL	DSO	2	3	4	5	6	7	8	9	10	11	12	13	14	15	16
1	IgM			<1:20		<1:20		1:20								
	IgG			<1:20		<1:20	<1:20	<1:20								
	Viremia			7.2		8.1	8.4	8.3								
2	IgM			<1:20	<1:20		1:20	1:20	1:20							
	IgG			<1:20	<1:20	<1:20	<1:20	1:20	1:40							
	Viremia			9.3	9.5	9.0	9.6	9.1	9.0							
3	IgM			<1:20	1:20				1:20		1:20			1:20		
	IgG			<1:20	1:20				1:20		1:20			1:80		
	Viremia			9.9	8.9				8.7		7.9			6.6		
4	IgM										1:160	1:160		1:160		
	IgG										1:320			1:640		
	Viremia										7.8	6.9		6.5		
5	IgM	1:20	1:20	1:80	1:160				<1:20							
	IgG		1:20	1:20	1:40				1:80	1:80						
	Viremia	7.6	7.0	7.1	6.8				5.1	6.4						
6	IgM				<1:20	<1:20	<1:20	<1:20	<1:20	<1:20	<1:20					
	IgG				<1:20	<1:20	<1:20	1:20	1:20	1:20	1:40					
	Viremia				8.3	8.2	7.6	8.2	8.2	8.4	8.6					
7	IgM			<1:20		<1:20	<1:20	<1:20	<1:20	<1:20		1:20	1:20		1:20	
	IgG			<1:20		<1:20	<1:20	1:20	1:20	1:20		1:40			1:160	
	Viremia			7.3		8.7	8.2	8.6	7.8	7.5		6.5	6.1		6.6	
8	IgM					<1:20	<1:20	<1:20	<1:20							
	IgG					<1:20	<1:20	<1:20	<1:20							
	Viremia					8.9	9.2	9.6	9.5							
9	IgM										<1:20	<1:20	<1:20			
	IgG										<1:20	<1:20	<1:20			
	Viremia										9.7	9.2	10			
10	IgM											1:40	1:40	1:40		1:80
	IgG											1:40	1:40	1:40		1:320
	Viremia											5.2	5.6	5.2		4.6
11	IgM					<1:20	<1:20	<1:20	<1:20	<1:20	<1:20					
	IgG					<1:20	<1:20	<1:20	<1:20	<1:20	<1:20					
	Viremia					9.4	9.0	8.2	8.1	8.2	8.5					
12	IgM						<1:20	<1:20	<1:20	<1:20	<1:20					
	IgG						1:20	<1:20	1:20	1:20	1:20					
	Viremia						8.5	8.8	8.5	8.6	8.2					
13	IgM						<1:20	<1:20			<1:20	1:10	<1:20	<1:20		
	IgG						<1:20	<1:20			1:20	1:20	1:20	1:20		
	Viremia						8.6	8.4			8.4	8.1	8.0	7.4		
